# Utility of the Global Respiratory Severity Score for predicting the need for respiratory support in infants with respiratory syncytial virus infection

**DOI:** 10.1371/journal.pone.0253532

**Published:** 2021-07-01

**Authors:** Jun Kubota, Daishi Hirano, Shiro Okabe, Kento Yamauchi, Rena Kimura, Haruka Numata, Takayuki Suzuki, Daisuke Kakegawa, Akira Ito

**Affiliations:** 1 Department of Pediatrics, Atsugi City Hospital, Kanagawa, Japan; 2 Department of Pediatrics, The Jikei University School of Medicine, Tokyo, Japan; Vanderbilt University Medical Center, UNITED STATES

## Abstract

**Background:**

Respiratory syncytial virus (RSV) is a common cause of acute respiratory infection in children. One of the most important strategies for treatment of an RSV infection is to decide whether the patient needs respiratory support. This study aimed to assess the validity and clinical benefit of the Global Respiratory Severity Score (GRSS) and the Wang bronchiolitis severity score (WBSS) for clinical decision-making regarding providing respiratory support (high-flow nasal cannula, nasal continuous positive airway pressure, or ventilator) in infants with an RSV infection.

**Study design and methods:**

This retrospective cohort study enrolled 250 infants aged under 10 months who were admitted to Atsugi City Hospital with an RSV infection between January 2012 and December 2019. The utility of these scores was evaluated for assessing the need for respiratory support through decision curve analysis by calculating the optimal GRSS and WBSS cut-offs for predicting the need for respiratory support.

**Results:**

Twenty-six infants (10.4%) received respiratory support. The optimal cut-offs for the GRSS and the WBSS were 4.52 and 7, respectively. Decision curve analysis suggested that the GRSS was a better predictive tool than the WBSS if the probability of needing respiratory support was 10–40%.

**Conclusions:**

The GRSS was clinically useful in determining the need for respiratory support in infants aged under 10 months with an RSV infection.

## Introduction

Respiratory syncytial virus (RSV) infection is a common cause of acute respiratory infection, particularly lower respiratory infection, in children [1, 2]. The severity of RSV infections ranges widely from mild to severe disease requiring ventilator support [3]. Most children are infected with RSV at least once by the time that they are two years old [4]. In infants with an RSV infection, the hospitalization rate is up to 20% [2, 3]. Since there is no specific treatment or vaccine and supportive care remains the primary treatment [5, 6], one of the most important strategies of treatment for an RSV infection is to decide whether the patient needs respiratory support [7, 8]. However, there is no standard scoring system for measuring the severity of an RSV infection. Some of the conventional scoring systems target different age groups or were developed for different purposes, such as measuring the outcomes of clinical trials and predicting the need for supportive care, including hospitalization, over relatively brief periods [9–11]. In 2017, the Global Respiratory Severity Score (GRSS) was developed as a specific scoring system to assess the requirement for hospitalization with RSV infection [9]. The GRSS includes both systematic and respiratory parameters, including age-specific respiratory rates for infants aged less than 10 months, and grades the overall severity over the course of illness. We hypothesized that the chronological severity score of the GRSS would be useful to guide decision-making regarding providing respiratory support. However, the GRSS has not been adequately validated. The bronchiolitis severity score developed by Wang et al. [12] (WBSS) has been recognized as a scoring system for lower respiratory infections for about 30 years. It is one of the most commonly used respiratory scoring systems in children, despite not being a specific scoring system for assessing the severity of the RSV infection, and has been used in previous studies [13–15]. Therefore, we chose the WBSS to compare with the GRSS.

The aim of this study was to assess the validity and clinical benefit of the GRSS and WBSS in order to guide decision-making in providing respiratory support for infants with RSV infections.

## Study design and methods

### Patients

We conducted a retrospective study at Atsugi City Hospital in Kanagawa, Japan. Patients aged <10 months who were admitted to the Department of Pediatrics at our hospital for treatment of an RSV infection between January 1, 2012 and December 31, 2019 were included in the study. In Japan, the number of RSV infections increased by 1.4-fold between 2012 and 2019; more cases were reported in the winter seasons throughout the study period (https://www.niid.go.jp/niid/ja/ydata/10071-report-jb2019.html). Only patients aged <10 months were included because Caserta et al. [9] used this age group to develop the GRSS. Patients were diagnosed with an RSV infection using rapid antigen-based tests. The Alere BinaxNOW RSV rapid test (Abbott Diagnostics, Abbott Park, IL, USA) and ALSONIC RSV test (Alfresa Pharma Corp., Osaka, Japan) were used from January 1, 2012 to March 31, 2019, and April 1, 2019 to December 31, 2019, respectively. Exclusion criteria were defined based on the exclusion criteria that Caserta et al. [9] used in the study in which the GRSS was developed. Hence, infants with any of the following conditions were excluded: a gestational age less than 36 weeks at birth; hospitalized for apnea only; high-risk conditions, such as chronic aspiration, congenital cardiac disease, immunosuppression, malignancy, and neurological conditions; indications for palivizumab prophylaxis; a history of admission in the neonatal intensive care unit; asthma; a history of wheezing; treatment with steroids; or a lack of the basic information needed to calculate the GRSS. The reason for excluding patients with a history of wheezing and treatment with steroids was to ensure that participants had an exclusive RSV infection without underlying diseases such as laryngomalacia, gastroesophageal reflux disease, and asthma. Infants taking steroids were excluded because steroids may mask the clinical signs used to calculate the GRSS and WBSS.

Starting respiratory support (with a high-flow nasal cannula, nasal continuous positive airway pressure, or a ventilator) was dependent on the pediatrician’s judgment based on clinical signs such as tachypnea, the presence of wheeze, rales/rhonchi, retractions, and respiratory acidemia on venous blood gas analysis.

### Study design

A retrospective cohort study was conducted and the medical records of each included patient was reviewed.

The primary predictors were the GRSS and the WBSS, and the primary outcome was the requirement for respiratory support.

Table 1 shows the parameters of the GRSS and the WBSS. The GRSS was calculated by entering ten parameters: age (months), oxygen saturation (%), respiratory rate (breaths/minute), general appearance, presence of wheeze, rales/rhonchi, retractions, cyanosis, lethargy, and poor air movement in the online calculator (available at: https://rprc.urmc.rochester.edu/app/AsPIRES/RSV-GRSS/) [9]. The WBSS was calculated using four parameters: respiratory rate in breaths/minute (<30: 0; 30–45: 1; 46–60: 2; >60: 3), wheezing (none: 0; terminal expiration or only with stethoscope: 1; entire expiration or audible on expiration without stethoscope: 2; inspiration and expiration without a stethoscope: 3), retractions (none: 0; intercostal only: 1; tracheosternal: 2; severe with nasal flaring: 3), and general condition (normal: 0; irritable, lethargic, feeding poorly: 3) [12]. With both scoring systems, higher scores were indicative of more severe signs of RSV infection. The GRSS and the WBSS were calculated using the worst signs in the day, but using the same signs to calculate each score.

**Table 1:** Parameters of each scoring system.

	Global Respiratory Severity Score	Wang bronchiolitis severity score
Age (months)	0–10	<24 months old
Oxygen saturation (%)	67–100	NA
Respiratory rate (breaths/min)	30–123	<30, 30–45, 46–60, >60
General appearance	Well, mild, moderate, severe, NA	Normal, abnormal (irritable, lethargic, poor feeding)
Wheezing present	Yes, no, NA	None, terminal expiration or only with stethoscope, entire expiration or audible on expiration without stethoscope or audible on inspiration and expiration, without a stethoscope
Rales/rhonchi present	Yes, no, NA	NA
Retractions present	Yes, no, NA	None, intercostal only, tracheosternal, severe with nasal flaring
Cyanosis present	Yes, no, NA	NA
Lethargy present	Yes, no, NA	NA
Poor air movement	Yes, no, NA	NA

In the Global Respiratory Severity Score, “NA” is allowed to be selected for only one of the seven parameters where it is listed as an option.

NA, not applicable

The patients were divided into two groups depending on whether they were provided with respiratory support. The GRSS and the WBSS were calculated based on the patient’s condition at the time of the assessment of the need for respiratory support. In infants who did not receive respiratory support, both scores were calculated using the worst measures during hospitalization.

The sick days were calculated using the onset of the presenting signs (rhinorrhea, cough, wheezing, fever, and lethargy) as the first day.

### Statistical analysis

Continuous variables are expressed as the median and interquartile range (IQR), and categorical variables are expressed as frequencies. Comparisons between the two groups were made using the Mann-Whitney *U-*test for continuous variables, and the chi-square or Fisher’s exact test for categorical variables.

The primary study objective was addressed in three steps. First, the optimal cut-off values for dichotomizing the GRSS and the WBSS were determined using the Youden index (the maximum value of [sensitivity–(1 –specificity)]) [16] based on the receiver-operating characteristic curve. Second, the groups that did and did not receive respiratory support were compared according to whether the GRSS and the WBSS were over the calculated optimal cut-off value. Last, we evaluated which score was more useful as an indicator of whether infants with an RSV infection need respiratory support using a decision curve analysis. Vickers et al. [17] explained the decision curve analysis as a method that “calculates a clinical ‘net benefit’ for one or more prediction models or diagnostic tests in comparison to default strategies of treating all or no patients.” The net benefit was calculated by using the formula:

Netbenefit=sensitivity×prevalence–(1–specificity)×(1–prevalence)×w

where *ѡ* is the odds at the threshold probability [17]. In this study, the net benefit represents the benefit of starting respiratory support, and the threshold probability represents the probability of starting respiratory support.

All analyses were performed using the Stata version 15.1 (StataCorp., College Station, TX, USA) software package. The statistical code for running the decision curve analysis was installed from the website (available at: www.decisioncurveanalysis.org). *P* values <0.05 were considered to be statistically significant.

### Ethical approval and informed consent

This study was conducted in accordance with the ethical principles of the Declaration of Helsinki and with the ethical guidelines for epidemiological studies issued by the Ministry of Health, Labour and Welfare, Japan. This study was approved by the Atsugi City Hospital Institutional Review Board (R2-03), which waived the requirement for obtaining informed consent from the patient’s guardian because the data were obtained retrospectively from the patients’ charts.

## Results

### Patient characteristics

A total of 366 infants aged <10 months were admitted to our hospital with an RSV infection during the study period. Of these infants, 250 (68.3%) fulfilled the inclusion criteria. The reasons for excluding 116 infants were: treatment with steroids (n = 33); history of wheezing (n = 31); incomplete medical records (n = 12); <36 weeks gestational age at birth (n = 10); unknown gestational age at birth (n = 8); congenital heart disease (n = 8); history of admission in the neonatal intensive care unit (n = 6); asthma (n = 2); immunosuppression (n = 1); neuromuscular disease (n = 1); congenital syphilis (n = 1); and others (n = 3). The characteristics of the included infants are shown in Table 2. The median age of the study patients was 2.9 months (IQR: 1.6–5.7 months), and 54.0% were male.

**Table 2:** Baseline characteristics of the study patients.

	Respiratory support (n = 26)	No respiratory support (n = 224)	*P*-value
Sex, n (%)			0.22
Male	17 (65.4)	118 (52.7)	
Female	9 (34.6)	106 (47.3)	
Age (months), median (IQR)	1.9 (1.0–3.2)	3.1 (1.7–5.8)	0.005
Gestational age at birth (week), median (IQR)	38.8 (38.0–39.6)	38.7 (38.0–39.9)	0.88
Sick days at the time of the worst score or on starting respiratory support, median (IQR)	5 (5–7)	5 (4–6)	0.13
GRSS, median (IQR)	4.86 (3.67–6.10)	2.79 (1.91–3.83)	<0.001
WBSS, median (IQR)	8 (7–9)	6 (5–7)	<0.001
High-flow nasal cannula, n (%)	9 (34.6)		
Nasal continuous positive airway pressure, n (%)	4 (15.4)		
Ventilator, n (%)	13 (50.0)		

GRSS, Global Respiratory Severity Score; IQR, interquartile range; WBSS, Wang bronchiolitis severity score

Twenty-six infants (10.4%) received respiratory support and 224 infants (89.6%) did not. The characteristics of both groups are shown in Table 2. The median sick days on starting respiratory support or the worst score was the fifth day for both groups (*P* = 0.13). The median age (months) of the infants who required respiratory support was significantly younger than that of the infants who did not require respiratory support (*P* = 0.005). The median GRSS and WBSS of the infants who required respiratory support were significantly higher than that of infants who did not require respiratory support (*P* < 0.001 and *P* < 0.001, respectively). However, none of the other variables considered differed significantly between the groups. Dataset is available as S1 Database.

### Optimal cut-off for dichotomization of the Global Respiratory Severity Score and the Wang bronchiolitis severity score

**Fig 1:**
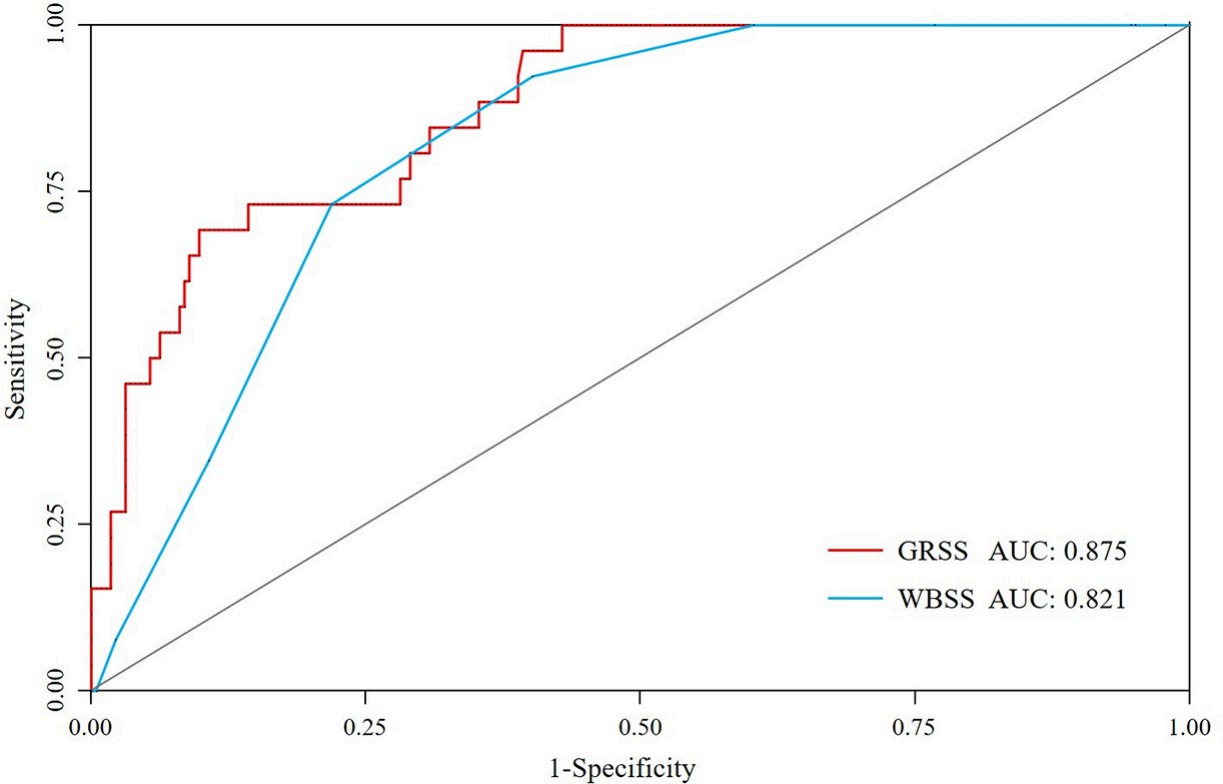
Receiver-operating characteristic curves of the Global Respiratory Severity Score and the Wang bronchiolitis severity score. The AUCs for the GRSS and the WBSS are 0.875 and 0.821, respectively, corresponding to cut-off values of ≥4.52 and ≥7, respectively.

As shown in Fig 1, the area under the curves of the GRSS and the WBSS were 0.875 and 0.821, respectively. The optimal cut-off for dichotomizing the scores, based on the receiver operating characteristic curve analysis, was 4.52 and 7 for the GRSS and the WBSS, respectively. Using these cut-offs, the GRSS and the WBSS had specificities of 90% and 60%, respectively, for predicting the need for respiratory support (Table 3).

**Table 3:** Values of the Global Respiratory Severity Score and the Wang bronchiolitis severity score.

	Cut-off	Sensitivity (95% CI)	Specificity (95% CI)	PPV (95% CI)	NPV (95% CI)
GRSS	4.52	0.69 (0.50–0.83)	0.90 (0.86–0.93)	0.45 (0.31–0.60)	0.96 (0.93–0.98)
WBSS	7	0.92 (0.76–0.98)	0.60 (0.53–0.66)	0.21 (0.15–0.29)	0.99 (0.95–1.00)

CI, confidence interval; GRSS, Global Respiratory Severity Score; NPV, negative predictive value; PPV, positive predictive value; WBSS, Wang bronchiolitis severity score

### Decision curve analysis for predicting the need for respiratory support using the Global Respiratory Severity Score and the Wang bronchiolitis severity score

**Fig 2:**
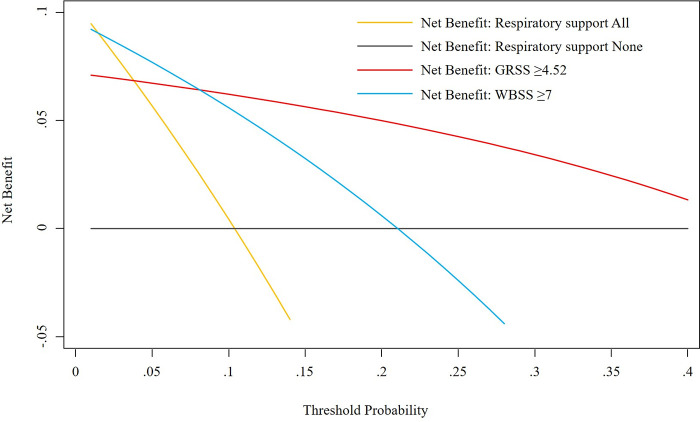
Decision curve analysis for predicting the requirement for respiratory support based on the Global Respiratory Severity Score and the Wang bronchiolitis severity score. The Y-axis measures the net benefit of respiratory support devices. The X-axis shows the probability of the need for respiratory support.

Fig 2 shows the decision curve analysis for predicting the need for respiratory support using the GRSS and the WBSS with cut-offs of 4.52 and 7, respectively. The GRSS was a better predictor than the WBSS if the probability of needing respiratory support was 10–40%. Conversely, the WBSS was a better predictor than the GRSS if the probability of starting respiratory support devices was <10%.

## Discussion

Our findings suggest that the GRSS is a potentially useful tool to enable clinicians to accurately predict the need for respiratory support among infants with an RSV infection. The optimal cut-off of the GRSS for predicting the need for respiratory support was 4.52.

Based on the decision curve analysis shown in Fig 2, we believe that the GRSS is more useful than the WBSS as a predictor of the need for respiratory support in clinical practice. This result was expected for three reasons. First, the American Academy of Pediatrics recommends supplemental oxygen administration in infants with bronchiolitis with an oxygen saturation < 90% [6, 18]. In addition, oxygen saturation level has been shown to be the most important predictor of the need for positive pressure ventilation and intensive treatment in hospitalized infants with bronchiolitis [19]. Therefore, it stands to reason that a scoring system for the severity of an RSV infection should include oxygen saturation, which is part of the GRSS, but not the WBSS. Second, in children, respiratory rates are very variable and change with age [20]. For example, the median, 10th percentile, and 90th percentile of the respiratory rates are 41, 30, and 56 breaths/minute in infants aged 0–3 months, compared with 29, 21, and 40 breaths/minute in children aged 18–24 months. The WBSS, which classifies the respiratory rates into four categories in children aged less than two years, is not adapted to these age-related changes in respiratory physiology, while the GRSS adjusts the respiratory rate according to age. Third, the GRSS provides a more comprehensive assessment of the respiratory condition than the WBSS because it includes three additional parameters: rales/rhonchi, cyanosis, and poor air movement. Therefore, it is understandable that the GRSS is a more accurate measure of the severity of an RSV infection compared to the WBSS.

The decision curve analysis can indicate which model is optimal to maximize the outcome of interest, while the area under the receiver operating characteristic curve focuses only on the predictive accuracy of a model [21]. In Fig 2, the net benefit represents the benefit of starting respiratory support, and the threshold probability represents the probability of starting respiratory support. The yellow line indicates that respiratory support should be initiated for all patients regardless of both scores. The red and blue lines indicate that respiratory support should be initiated based on the GRSS or WBSS, respectively. At each threshold probability (probability of starting respiratory support), it can be verified which line indicates the highest net benefit. The decision curve analysis finding that the GRSS is a good indicator of a 10–40% chance of needing respiratory support is consistent with evidence from other studies. In a post hoc analysis of a national database an average of 17.6% of children aged less than two years with a hospitalized RSV infection between 2004 and 2013, including those admitted to an intensive care unit, required respiratory support [22]. In a prospective multicenter observational study, the median proportion of children with bronchiolitis aged less than two years who required respiratory support ranged from 15 to 26% [23]. Of the participants in the study, 70% had an RSV infection [23].

Pediatricians should determine the need for respiratory support as soon as possible because early initiation of respiratory support is the most important treatment strategy for children with bronchiolitis, including an RSV infection [7]. Between 2000 and 2016, the use of mechanical ventilation and hospital costs significantly increased in children with bronchiolitis aged less than two years despite the overall decline in the hospitalization rate for bronchiolitis [24, 25]. Therefore, chronological use of the GRSS, which has the specificity (90%) and negative predictive value (96%) (Table 3) to guide decisions regarding the initiation of respiratory support in infants with an RSV infection could reduce the burden of unnecessary treatment and save healthcare costs.

This study had several limitations. First, the GRSS is for use in infants aged less than 10 months, so it does not provide guidance for older children. However, the rate of hospitalization with RSV infections in infants aged less than 5 months is about 2–5 times higher than those aged more than 5 months [1, 26]. In this study, children with respiratory support were significantly younger than those without respiratory support. Therefore, the age criterion of the GRSS is not a major limitation. The results of this study might have been better for the GRSS than the WBSS because the exclusion criteria for this study were based on the exclusion criteria used to develop the GRSS. When the WBSS was developed, patients who required 35% or more of inspired oxygen were excluded [12], so the WBSS is not well suited to scoring patients with severe disease. As more than 80% of infants hospitalized with an RSV infection do not have any underlying conditions and are otherwise healthy [27], the GRSS exclusion criteria are likely to be more representative than the WBSS exclusion criteria, of infants hospitalized with RSV infection. In future, further studies are needed to expand the criteria of the GRSS. Second, this study was conducted at a single facility. Third, the admission of patients with an RSV infection and starting respiratory support was dependent on the pediatrician’s judgment based on clinical signs. There was a limited turnover of pediatricians during the study period, hence the criteria for admission and starting respiratory support are unlikely to have changed during the study period. Fourth, not all infants aged less than 10 months old were tested using rapid diagnostic testing at admission because this was undertaken based on respiratory symptoms and the RSV epidemic. Fifth, rapid antigen diagnostic testing, which has a lower sensitivity and specificity than polymerase chain reaction, was used. However, in view of its relatively low cost, we considered that the diagnostic accuracy of rapid antigen diagnostic testing was adequate for clinical situations. Lastly, the GRSS and the WBSS were evaluated by selecting the worst findings in the day because this study was a retrospective study. However, they were scored at the time of medical examination in actual clinical settings. To address these limitations, the GRSS should be scored using the findings at the time of medical examination in a multicenter study.

In conclusion, the GRSS was clinically useful in determining the need for respiratory support in infants aged under 10 months with an RSV infection.

## Supporting information

S1 Database (XLSX)
